# Prescription Department

**Published:** 1892-03

**Authors:** 


SPERMATORRHŒA.—

In stubborn cases of spermatorrhœa combine ergot with neurosine as in the following formula:

R Fl. ext. ergot, oz. j.
Neurosine, oz. iv.

M. Sig. Teaspoonful in water three times a day and at bedtime.

When a chalybeate or other tonic is desired, together with a nervine, the following will prove eligible:

R Ferri et quinæ ammon cit., dr j.

Neurosine, oz. vi.

M. Sig. Teaspoonful three times daily.

AMENORRHŒA WITH GREAT DEBILITY

Stearnes' cascara aromatic, 1 fl. oz.
Simple elixir, 2 fl. oz.
Fl. ext. of berberis aquifolium 2 fl. oz.

M. Sig. Dose, one-half to one teaspoonful three times a day.

DYSMENORRHŒA WITH HABITUAL CONSTIPATION.—

Stearns' cascara aromatic, 1 fl. oz.
Simple Elixir, 2 fl. oz.
Syr. of Sarsaparilla compound, 1 fl. oz.

Mix. Sig. Tesspoonful three times a day before meals.

JAUNDICE.—

R Hydrarg. cor.chlor. per dose, 1-16 grain.
Potassi chloridi, per dose, 2 grains
Tinct. cinchonæ, 5 ounces.
Tinct. hyoscyami, 3 ounces.

M. Sig. Drachm four times a day.

ANTISEPTIC ADHESIVE OIL.—

R Zinc oxidi, 5 grains.
Zinc chloridi, 20 grains.
Gelatinæ, 5 drachms.
Listerine 7½ drachms. M.

This dressing protects the surface of wounds and dispenses with the use of bandages after operations. It is especially of service for the dressing of wounds on the face.—*Ex.*

DISSOLVING DIPHTHERITIC MEMBRANES.—

Caldwell is stated to recommend the following solution for this purpose:

R Papain, 2½ drachms.
Hydronaphthol, 2 grains.
Acid muriatic, 15 drops.
Aquæ destillat, 3 ounces.
Glycerini, 2 drachms.

M. Sig. Apply to the affected parts every half hour by means of an atomizer.—*Medical News.*

Subinvolution of the Uterus.—

Dr. B. C. Hirst suggests the following as the best combination to use:

℞ Strychninæ sulphatis, gr. 1-20.
Quininæ sulphatis, gr. ij.
Ext. ergotæ, gr. j.

M. Et. ft. pil. No. 1. Sig. At one dose.—*Medical World.*

Broncaial Catarrh.—

℞ Acetat. scillæ, oz. ss.
Ext. ipecac, f. dr. ss.
Tr. opii deod., dr. j.
Syrup tolu, dr. x.

M. Sig. A teaspoonful every two, three or four hours.—Bartholow.—*Ex.*

For a case of idiopathic epilepsy in a child of three years of age, Dr. Edwin E. Graham said that the treatment by the mixed bromides was the best, and prescribed the following:

℞ Potassii bromid., gr. iij.
Sodii bromid., gr. ij.
Ammonii bromid., gr. j.

M. Sig. Four times a day.—*Ex.*

Professor Pavin recommended to the class the following formula for making a palatable preparation of cod liver oil, which he had found that children would take very readily:

℞ Olei morrhuæ, f. oz. viij.
Olei gaultheriæ, f. dr. j.
Calcii phosphatis, gr. cclvj.
Pulv. acaciæ.
Sacchari albi, aa oz ij.
Aquæ, q. s. ad Oj. M.

—*Coll. and Clinic.*

Putrid Sore Throat.—Late J. Milner Fothergill, M. D., London, England:

℞ Tr. myrrhæ.
Aceti, aa oz. ij.
Mellis, oz. j.
Infusion serpentaria, O iiss.

M. fiat gargarisma.—*Ex.*

Gonorrhœa—

℞ Chloral hydrat.
Zinc sulph., aa gr. iv.
Aquæ, oz. iv.

Use one-fourth contents of the bottle a. m. and p. m. as an injection.—*Ex.*

As a carminative, the following mixture is excellent:

℞ Tinct. calumbæ, dr. iij.
Spts. ammon. aromat., dr. iss.
Tr. cardamomi com. q. s. u. f iij oz.

M. Sig. Oz. ss. *pro re nata.*—*Ex.*

